# Enhanced pain facilitation rather than impaired pain inhibition in burning mouth syndrome female patients

**DOI:** 10.1186/s10194-022-01516-7

**Published:** 2022-11-18

**Authors:** Christelle Gremeau-Richard, Paul Pionchon, Aurélien Mulliez, Christian Dualé, Radhouane Dallel

**Affiliations:** 1 Université Clermont Auvergne, CHU Clermont-Ferrand, Inserm, Neuro-Dol, Faculté de Chirurgie Dentaire, 2 Rue de Braga, 63100 Clermont-Ferrand, France; 2 Present Address: Faculté de Chirurgie Dentaire, 2 Rue de Braga, 63100 Clermont-Ferrand, France

**Keywords:** Orofacial pain, Diffuse Noxious Inhibitory Control, Temporal summation, Heat, Hyperalgesia, Depression

## Abstract

**Background:**

Deficient endogenous pain modulation has been implicated in the development and exacerbation of chronic orofacial pain. To date, relatively little is known regarding the function of the endogenous pain modulation in patients with burning mouth syndrome (BMS). This case–control study investigated endogenous pain modulation in women with BMS.

**Methods:**

Conditioned pain modulation (CPM) was assessed upon temporal summation (TSP) of thermal pain. Forty female subjects, 20 BMS patients and 20 age-matched control subjects, were included in a 2 session-protocol. Mechanical and thermal pain thresholds were measured on the forearm and hand. TSP was obtained using repetitive laser-evoked thermal stimuli applied on the non-dominant hand, at an intensity yielding to moderate pain. During TSP, CPM was produced by immersing the contralateral foot in a water bath at painful cold (8 °C) temperature. In control conditions, the foot was immersed in a water bath at not painful (30 °C) temperature.

**Results:**

BMS was not associated with any impairment in thermal as well as mechanical extracephalic pain thresholds. TSP and CPM efficacy were similar in BMS patients and control subjects. However, BMS patients exhibited enhanced extracephalic heat hyperalgesia.

**Conclusion:**

This study reveals that there is no impairment of endogenous pain inhibition mechanisms in BMS patients, but rather an increase in pain facilitation.

## Key findings

• BMS was not associated with any impairment in thermal as well as mechanical extracephalic pain thresholds.
• TSP and CPM efficacy were similar in BMS patients and control subjects.
• BMS patients exhibited enhanced extracephalic heat hyperalgesia

## Background

Burning mouth syndrome (BMS) is a chronic and spontaneously non-remitting oral pain without any identifiable local anatomical lesion or laboratory finding [1]. The pathogenesis and etiology of BMS are still unknown. Although it was initially classified as a psychalgic pain [2], recent evidence suggests that BMS is rather a neuropathic disorder [1]. However, the very cause of the neuropathic changes is still unclear. Combined dysregulations of adrenal, gonadal and neuroactive steroids, dysfunction of gustatory and somatic afferents have been suggested to contribute to the pathogenesis of BMS [1]. Interestingly, BMS often coexists with other syndromes referred to as central sensitivity syndromes [3] such as temporo-mandibular disorder (TMD), fibromyalgia or visceral pain [4, 5]. BMS might thus share common mechanisms with these chronic pain syndromes, including abnormal endogenous pain modulation [6, 7]. One of the major endogenous pain inhibitory systems is conditioned pain modulation (CPM; formerly diffuse noxious inhibitory controls), a powerful general endogenous analgesic mechanism which can completely inhibit incoming nociceptor signals at the primary synapse [8]. CPM is believed to play an important role in the development and exacerbation of chronic pain, because dysfunction of CPM is associated with a shift in balance between pain facilitation and pain inhibition. In many (but not all) patients with central sensitization, CPM is less efficacious [9]. Although an association between deficient CPM and the development of BMS was found in a previous study [10], here, we reassessed CPM in BMS patients using a brand new experimental protocol [11]. The primary endpoint of this exploratory case–control study was to evidence any alteration of CPM in BMS patients.

## Methods

### Study design

This is a case–control study conducted in the Clinical Investigation Centre of Clermont-Ferrand University Hospital (France). The study conformed to the STROBE guidelines for case–control studies. The study was performed after approval was obtained from the referent Ethics Committee “CCP Sud-Est VI”. Standard written informed consent was obtained from all participants, according to the 1964 declaration of Helsinki. The study was registered as N°2010-A00403-36.

### Participants

The sample size calculation was based on pilot data obtained in eight healthy subjects from the Clermont-Ferrand center, in which CPM decreased the test heat pain by 2.6 ± 1.8. Expecting that the CPM in BMS patients would be decreased compared to controls as it is the case in fibromyalgia patients [12], the sample size was calculated to allow for the identification of a difference of 1.7 points (65% reduction) with a standard deviation of 1.8. With a statistical power of 80% and a two-tailed type I error of 5%, 18 patients per group were found to be necessary. We decided to include 20 subjects per group to increase the power of the study.

The patients were recruited from the Clermont-Ferrand Hospital Orofacial Pain Clinic. Identification of the patients was carried out taking into account the inclusion/exclusion criteria reported below.

#### Inclusion criteria

BMS group: females patient over 40 year-old; daily presence of localized burning sensation in the oral mucosa during all or part of the day lasting for longer than 3 months; no paroxysm and not following any unilateral nerve trajectory; no clinical oral mucosal alterations; pain intensity ≥ 3 on a Visual Analogue Scale (VAS).

For all participants: subjects must have given their written consent.

#### Exclusion criteria

BMS group: report of pain and/or burning in the mouth of known origin (cancer, infectious, traumatic, iron deficiency anemia, diabetes); recent change in current medication. Unknown local or systemic pathologies were ruled out thanks to the medical history and blood sampling looking for iron deficiencies, vitamins, diabetes or thyroid dysfunction.

For all participants: taking one of the following antidepressants: venlafaxine, duloxetine, tricyclic antidepressants or bupropion; current medication with neuroleptics; co-morbidity with another ongoing chronic pain (*e.g*. fibromyalgia, chronic low back pain, chronic pelvic pain, tension headache, migraine, irritable bowel syndrome, temporomandibular disorder); progressive cardiovascular disease; subject not cooperating or unable to speak or read French fluently, or to understand a pain scale; major cognitive or incapacitating disorder.

The control group consisted of 20 females, matched for age, who were recruited among patients attending the Oral Medicine Clinic for identifiable organic causes such as decayed tooth, dental treatment and other mucosal diseases. Only women were included in the study, since BMS is mostly a woman’s pathology and CPM display sex specific features [6].

### Questionnaires

Standard demographic information, duration of complaint, health status, medical use and pain medications used before the examination were collected. At the same time, all subjects also completed a series of self-report questionnaires including the Beck Depression Inventory (BDI) short form, the State-Trait Anxiety Inventory (STAI) and the Pain Catastrophizing Scale (PCS).

### Pain intensity ratings

The burning pain intensity in the past week was rated using both the 0–10 cm visual analogue scale (VAS) and the 4-point-(none, mild, moderate and severe)-verbal rating scale (VRS).

### Pain threshold measurements

Explorations were conducted in quiet conditions and at constant temperature (23 °C). For measurement of mechanical pain thresholds (MPTs), punctuate stimuli were applied on the forearm using an electronic algometer (electronic von Frey, Bioseb, France). The strain gauge was connected to a plastic sterile cone (Eppendorf, Hamburg, Germany), the tip of which was applied perpendicularly to the studied skin area. The punctate pressure was gradually increased with a constant slope under visual control of the pressure value up to the detection of MPT. Threshold was defined as the lowest pressure that produced a sensation of pain. The results of three separate consecutive measurements at different points in the testing area were averaged to establish the MPT value. The applied pressure could not exceed 500 g, which was the default threshold if pain was not elicited at this level.

Measurement of heat pain and tolerance thresholds were performed using a thermotester (SENSELab®, Somedic, Sweden) on the skin of the non-dominant forearm. The dimensions of the skin thermode were 25 × 50 mm. The baseline temperature was set at 32 °C, the maximum temperature was 52 °C and the temperature change rate was set at 0.5 and 1.0 °C.s^–1^, respectively for pain and tolerance threshold measurement. The patient pushed a stop-button when the threshold was reached and there was an interval of five seconds between repeated thermal stimuli. The whole procedure was repeated five times and the average of five thresholds was taken. In pre-experimental explanations given to patients, particular emphasis was placed on the concept of pain threshold as opposed to tolerance threshold, in order to avoid bias due to patient fear. Immediate pain intensity was scored by the patient on a 0–10 cm visual analogue scale (VAS).

### Conditioned pain modulation measurements

To explore the subjects’ individual CPM potency, the temporal summation of pain (TSP) evoked by a stimulation of the back of the non-dominant hand by a YAP laser stimulator (fiber-optic guidance, diameter 5 mm, duration 2 ms) was used as test stimulus. A stimulator with optic-fiber guidance was placed on a skin area of about 6 cm^2^ at the dorsal aspect of the hand. Cutaneous heat stimuli were applied in incremental intensities in steps of 0.25 J, starting at a minimum of 0.75 J and reaching a maximum of 2 J. After determination of the pain threshold, the laser energy that was able to produce a pain intensity between 3–6 on 0–10 VAS was determined (~ 10 min before CPM assessment). On the one hand, such intensity was needed to elicit sufficient pain to observe a CPM effect, but, on the other hand, not too strong, for ethical reasons. Thereafter, the stimulation at this same intensity was applied repeatedly (from 1st to 45th) every 40 s for 30 min. Thus, each patient received a series of 45 stimulations applied at constant intensity. Subjects were asked to score pain on a 0–10 VAS scale after each block of stimulation. The laser beam was moved (~ 1 cm) between each block to avoid skin lesions.

The conditioning stimulus consisted in the immersion of the contralateral foot into a water bath at noxious cold temperature (8 °C), between the 10^th^ and 20^th^ min of the experiment. In control experiments that were performed on a different day in the same subjects, the water bath temperature was neutral (30 °C). In all cases, water was constantly re-circulated to prevent laminar warming around the immersed foot. All subjects were asked to rate the cold pain on a 0–10 VAS scale immediately at the end of immersion. The order CPM *vs.* control experiments was assigned randomly.

The responses for each block in each condition were plotted over time. The first 15 responses were used to study the unconditioned responses (1st to 15th), the next 15 ones (16th to 30th) to study CPM and the 15 last ones (31st to 45th) to study the post-effects of CPM.

### Statistical analysis

Statistics were computed with STATA V12 (Stata Corp, College Station, TX, USA). Results were expressed as mean ± standard deviation or median (interquartile range) and as frequencies (percentage). Groups were compared using Chi square test (or Fisher’s exact test when appropriate) for categorical data, and using Student t-test (or Mann–Whitney when data not normal) test depending on data distribution for continuous data. Normality was assessed graphically and using Shapiro–Wilk's test. Relationships between the pain intensity and BDI, STAI, and PCS scores were assessed by Pearson’s correlation coefficient (or Spearman’s when data were not normal). Intra-groups comparisons of pain at discrete time points were performed using Students paired t-test.

Pain perception was analyzed using linear mixed models with random subject intercept. Groups, time and foot water immersion were tested as fixed effects. Those methods were developed for temporal subdivision (control, water immersion and post-water immersion periods) in order to test: 1) the session effect, 2) the effect of the repeated painful stimulation, 3) the water immersion effect and the post-water immersion effect. Those analyzes were repeated intra and inter-groups. Moreover, for the 1) and 2) we adjusted for the mean pain of the 15 first blocks (= baseline adjustment). All tests were two-sided and a *P* value < 5% was considered statistically significant.

## Results

### Study population

The control participants were age- and gender-matched with the BMS patients. The clinical characteristics of the BMS patients are displayed in Table 1. No differences were found between groups, except that BMS patients were more anxious than controls. The depression scores of all participants were within the normal range. The mechanical, heat and laser pain thresholds did not differ between BMS patients and healthy controls (Table 2).

**Table 1:** Demographic and clinical characteristic of BMS patients and control subjects

Variables	BMS (*n* = 20)	Controls (*n* = 20)	*P values*
Age, mean (SD), range	64.3 (8.2), 49–80	62.4 (7.6), 47–77	0.453
Menopause, n (%)	19 (95.0)	19 (95.0)	1.000
Age of menopause, years (SD)	48 (6.3)	50.9 (2.6)	0.257
Hormonal treatment, n (%)	2 (10.0)	2 (10.0)	1.000
Genital burning, n (%)	3 (15.0)	0 (0)	0.230
Hysterectomy, n (%)	6 (30.0)	2 (10.0)	0.232
Oophorectomy, n (%)	3 (1.5)	0 (0)	0.230
Beck Depression Inventory score, median (IQR)	16 [16.0–21.5]	14 [13.0–18.0]	0.054
State Trait Anxiety Inventory score, mean (SD)	51.9 ± 3.6	48.6 ± 3.9	0.009*
Pain Catastrophizing Scale (Sullivan scale), mean (SD)	18.5 ± 13.2		
Characteristics of intraoral pain			
Duration of complaint, years, median (IQR)	3.0 [3.0–4.0]		
Pain expressed on VAS, median (IQR)	5.8 [4.7–6.9]		
Pain expressed on VRS, n (%)	mild: 2 (11.1) moderate: 14 (77.8) severe: 2 (11.1)		
Pain locations, n (%)	tongue 20 (100) lip 10 (50.0) palate10 (50.0)		

Abbreviations: *VAS* Visual analogue scale, *VRS* Verbal rating scale, *SD* Standard deviation, *IQR* Interquartile range

^*^P value is from two-tailed Student's unpaired t-test

**Table 2:** Mechanical and thermal pain thresholds and pain rating

Parameters, mean (SD)	BMS (*n* = 20)	Controls (*n* = 20)	*P values*
Mechanical pain threshold (forearm) (g)	175.2 ± 54.3	187.3 ± 79.3	0.579
Heat pain threshold (forearm) (°C)	42.6 ± 4.6	44.0 ± 4.1	0.310
Heat pain tolerance threshold (forearm) (°C)	47.8 ± 3.1	47.7 ± 3.1	0.957
Laser pain threshold (back of the hand) (J)	1.39 ± 0.33	1.38 ± 0.22	0.906
Temporal summation of pain (VAS)	13.6 ± 18.2	19.9 ± 22.4	0.338
Sum of pain intensity between 1^st^-15^th^	726.5 ± 168.3	585.9 ± 185.8	0.016*

Abbreviations: *g* gramme, *J* Joule, *SD* Standard deviation

^*^P value is from two-tailed Student's unpaired t-test

### Temporal summation of pain

**Fig. 1:**
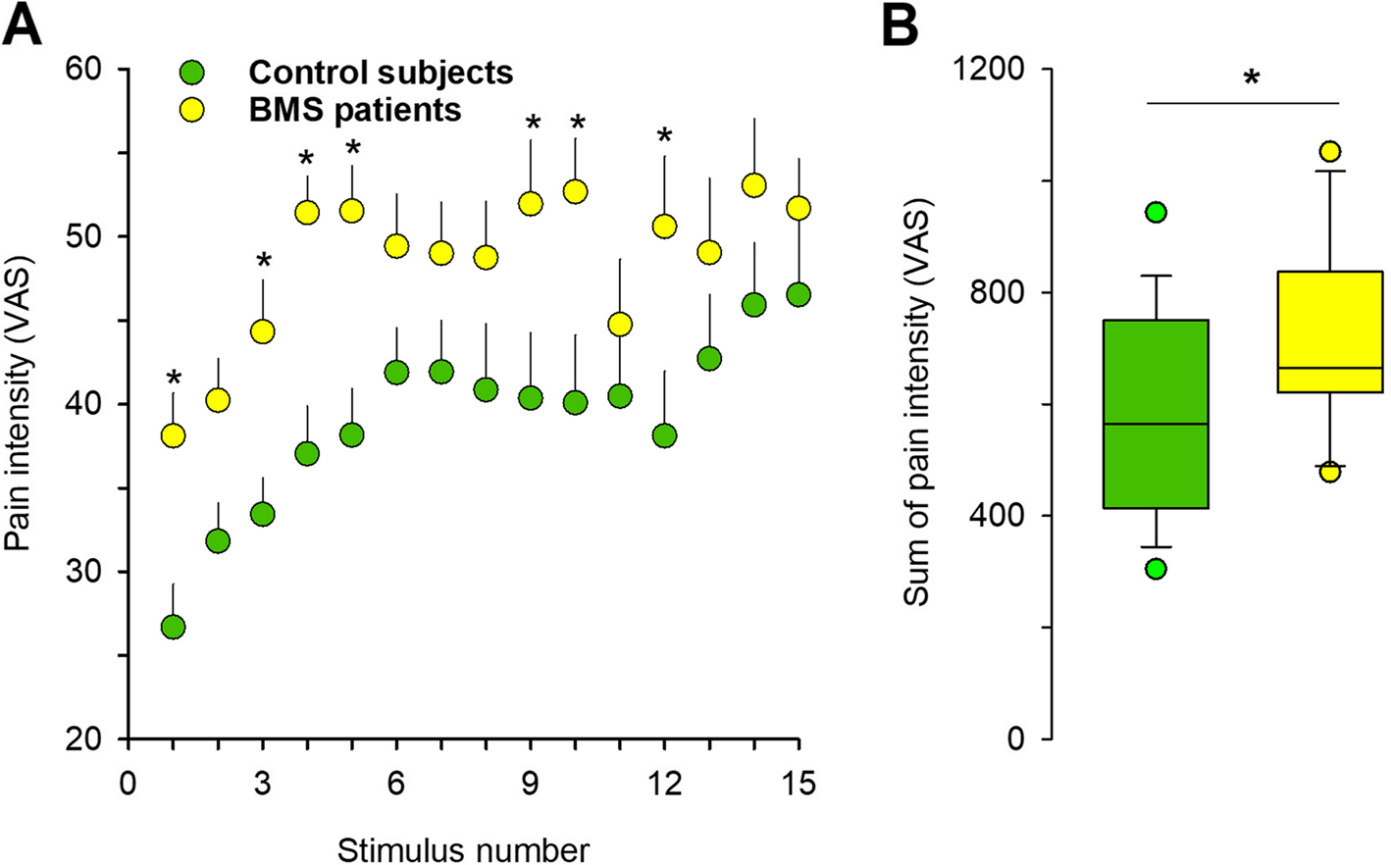
Temporal summation of pain in burning mouth syndrome (BMS) patients and control subjects. **A** Time course of pain perception evoked by repetitive laser painful stimuli applied of the hand in control subjects, and BMS patients. Testing sessions consisted of a series of 15 blocks of 4 thermal test stimuli (Nd:YAP laser stimulator) that were delivered at 0.2 Hz on the back of the non-dominant hand, repeated every 40 s for 10 min. Mean pain ratings (± SEM) are shown from the first to the 15th stimulation. *Linear mixed model: *P* = 0.012 for group, *P* < 0.001 for time, *P* = 0.514 for time × group. **B** Sum of pain intensity between 1^st^-15^th^.^.^ *P value is from two-tailed Student's unpaired t-test

Following repetitive laser-mediated thermal stimuli, pain intensity progressively increased in both groups, showing TSP (Fig. 1) Pain intensity increased from the first to the 15^th^ block of stimulation in control subjects (paired t test, t = 4.94, df = 39, *P* < 0.001), from 26.7 ± 2.7 to 46.5 ± 4.8, as well as in BMS patients (paired student t-test, t = 3.45, df = 39, *P* = 0.001), from 38.1 ± 2.6 to 51.7 ± 3.1. Moreover, while the magnitude of TSP – as calculated by the difference between the last and the first pain rating – did not differ between BMS patients and control subjects (Student's t-test, t = 1.11, df = 78, *P* = 0.269), the sum of pain intensities between the 1^st^ and the 15^th^ block of stimuli was greater in BMS patients than control subjects (Fig. 1). Thus, BMS patients demonstrated significantly greater heat hyperalgesia than control subjects (Linear mixed model: *P* = 0.012 for group, *P* < 0.001 for time, *P* = 0.514 for time × group.), while there was no difference in TSP.

As BMS patients exhibited higher STAI scores than control subjects, we tested whether the severities of anxiety, on the one hand, and pain (between the 1st and 15th block of stimuli), on the other hand, are correlated. There was no correlation between STAI scores and pain intensities in the control subjects (chi^2^ = 0.02, df = 1, *p* = 0.893), as well as BMS patients (chi^2^ = 1.51, df = 1, *p* = 0.219). We also tested whether STAI scores (which are higher in BMS patients than in control subjects) might affect the group effect, by assessing the group x STAI interaction. We found no significant interaction (chi^2^ = 0.56, df = 1, *p* = 0.456).

### Conditioned pain modulation

In the present study, we performed two analyses. First, we assessed the intensity of pain evoked by the laser stimulation before (1^st^-15^th^ stimulations), during (effect, 16^th^-30^th^ stimulations) and after (post-effect, 31^st^ -45^th^ stimulations) immersing the contralateral foot into a water bath at painful cold (8 °C) temperature. Second, we also compared the intensity of laser stimulation-evoked pain during (effect, 16^th^-30^th^ stimulations) and after (post-effect, 31^st^-45^th^ stimulations) application of the painful conditioning stimulation (water at 8 °C, 16^th^-30^th^ stimulations) with those during and after application of a non-painful conditioning stimulation (water at 30 °C).

**Fig. 2:**
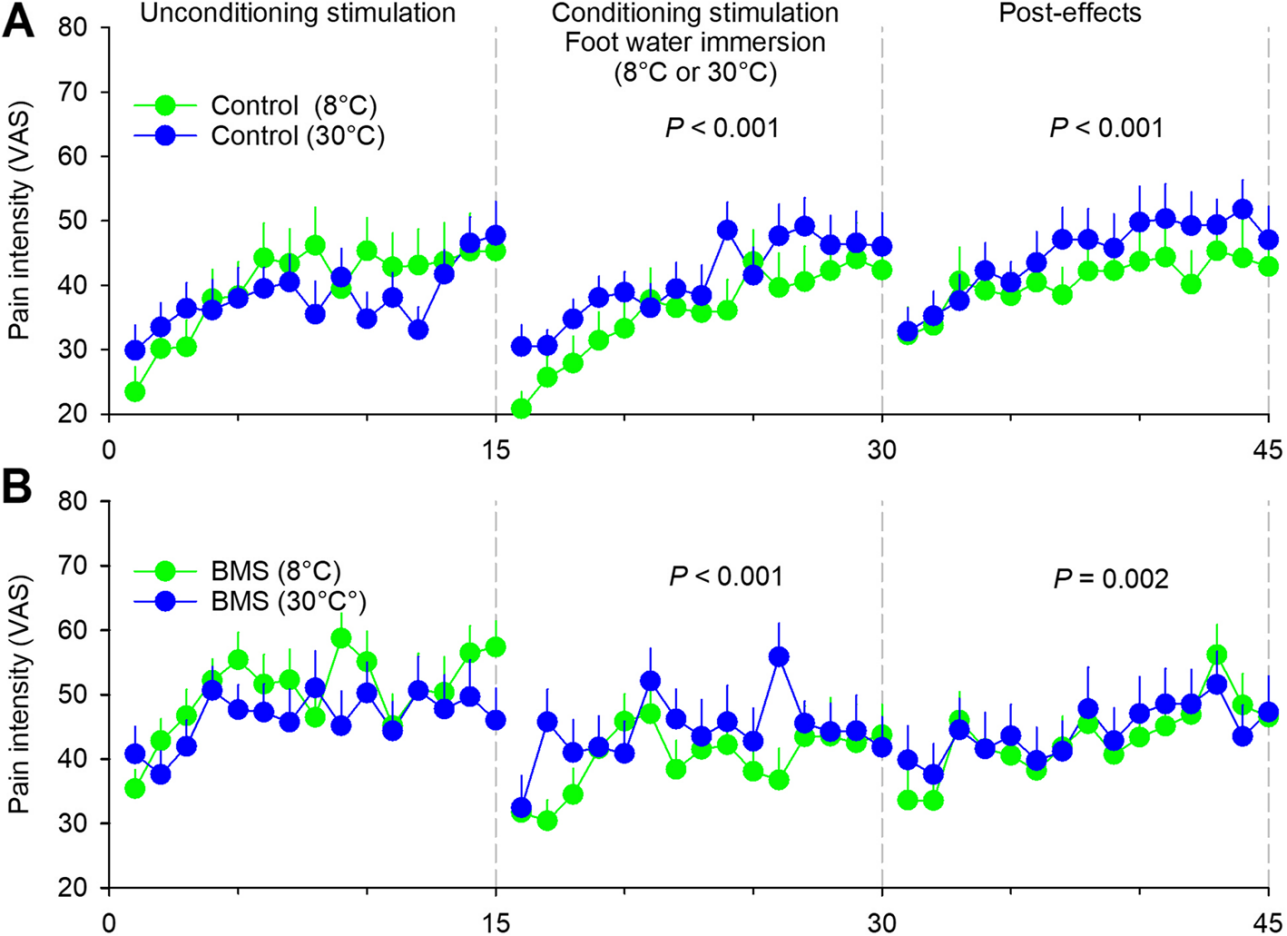
Time course of pain perception evoked by repetitive painful stimuli of the hand before (1st to 15th), during (16th to 30th), and after (31st to 45th) application of non-painful (in yellow) or painful (in green) conditioning thermal stimulation on the contralateral foot in (**A**) control subjects and (**B**) burning mouth syndrome patients. To assess CPM, blocks of 4 thermal test stimuli (Nd:YAP laser stimulator) were delivered at 0.2 Hz on the back of the non-dominant hand, repeated every 40 s for 30 min, at an intensity producing a pain intensity between 3–6 on 0–10 VAS. Between the 10th and 20th min (16th to 30th), the contralateral foot was immersed into a water bath at non-painful (30 °C) or painful cold (8 °C) temperature. A linear mixed model was used to explore the effects of CPM. *P* values indicate the differences between the non-painful and painful conditioning thermal stimulation

In control subjects, laser-evoked pain was depressed during (time effect, chi^2^ = 119, df = 14, *P* < 0.001) and after (time effect, chi^2^ = 58.7, df = 14, *P* < 0.001) the application of the painful conditioning stimulation on the contralateral foot (Fig. 2A). Compared to the non-painful conditioning stimulation, the magnitude of CPM was higher during (cold-water effect, chi^2^ = 29, df = 1, *P* < 0.001) and after (cold-water post-effect, chi^2^ = 20, df = 1, *P* < 0.001) the application of the painful conditioning stimulation of the contralateral foot.

In BMS patients, laser-evoked pain was also depressed during (time effect, chi^2^ = 34.1, df = 14, *P* = 0.002) and after (time effect, chi^2^ = 42.6, df = 14, *P* < 0.001) the application of the painful conditioning stimulation on the contralateral foot (Fig. 2B). Compared to non-painful conditioning stimulation, the magnitude of CPM was higher during (cold-water effect, chi^2^ = 19.5, df = 1, *P* < 0.001) and after (cold water post-effect, chi^2^ = 9.47, df = 1, *P* = 0.002) the conditioning stimulation. However, there was no group effect (patients *vs.* controls) on the CPM, either during (interaction group x cold-water effect, chi^2^ = 0.04, df = 1, *P* = 0.837) or after (interaction group x cold water effect, chi^2^ = 0.73, df = 1, *P* = 0.392) the conditioning stimulation.

## Discussion

In this study, we investigated whether impaired endogenous pain modulation could be part of the mechanisms underlying BMS in women. We found no changes in CPM efficiency in BMS patients, no changes in TSP, while neither thermal nor mechanical extracephalic pain thresholds were altered. However, the sensory profile of BMS patients was characterized by enhanced extracephalic heat hyperalgesia.

CPM is a powerful endogenous analgesic mechanism whereby a nociceptive stimulation applied to a given body location reduces the percept and brain responses elicited by noxious (test) stimuli delivered at a remote body location [6, 8]. Dysfunction of CPM is assumed to shift the balance between pain facilitation and inhibition toward facilitation and the development of chronic pain [6]. Here, we tested the hypothesis that abnormal CPM may be part of the pain mechanisms in BMS. However, in contrast to a previous study [10], this was not the case, since BMS patients were able to normally recruit their endogenous pain inhibitory system in our experimental setting. A similar hypothesis has been raised regarding the pathophysiology of other chronic orofacial pain conditions, but CPM has not always been reported to be impaired [13, 14] and appeared normal in some studies [7, 15–17] Such contradictory results – due in part to differences in methods used to assess CPM [6]—and the lack of aggregate analysis of the literature prevent any solid conclusion about the function of endogenous pain modulation in patients with chronic orofacial pain [7].

TSP, when assessed in response to repetitive laser-evoked thermal stimuli [11], was similar in BMS patients and control subjects. Similarly, a previous study, using intradermal electrical stimulation of the chin, found no difference in TPS between BMS and controls [10]. This is consistent with previous findings in other chronic orofacial pain syndromes, such as TMD, where TSP was normal when assessed with thermal stimuli [18–22]. In such patients though, TSP could be found altered [17, 23], when assessed with mechanical stimuli, as it sometimes increased [20]. Since thermal TSP relies, at least in part, on the activation of N-methyl-D-aspartate (NMDA) receptors, and thermal TSP is not altered in BMS patients, it seems likely that NMDA receptors do not play a critical role in the pathophysiology of BMS. Similar mechanistic suggestions have been made about patients suffering from persistent post-endodontic pain [14].

Despite normal TSP, BMS patients showed greater extracephalic heat hyperalgesia than control subjects. An enhanced heat hyperalgesia seems common in patients with chronic, either cephalic [20, 21] or extracephalic [24], pain. In all these conditions, the presence of hyperalgesia suggests a facilitation of pain mechanisms in BMS patients. In addition, hyperalgesia has been shown to be predictive of poor outcomes in some chronic pain conditions [25], and this may also apply to BMS.

Although we cannot exclude the possibility that the enhanced heat hyperalgesia in BMS patients results from peripheral mechanisms, several observations rather suggest a central contribution. First, neither heat pain thresholds nor MPTs were reduced in the BMS group, showing that thermal and mechanical nociceptors were not sensitized. Second, that That hyperalgesia was observed in a healthy area (hand), remotely located from the painful site (mouth), suggests that it is a secondary hyperalgesia. There is ample evidence for secondary hyperalgesia resulting, at least in part, from a facilitation of nociceptive transmission – i.e. central sensitization – within the dorsal horn [26]. Finally, since CPM efficiency was similar in BMS patients and controls subjects, a reduction in CPM cannot explain the enhanced heat hyperalgesia that may rather relies on increased pain facilitating mechanisms.

Prevalence of psychological disorders, anxiety especially, is high in BMS patients, but its role in the pathogenesis of BMS remains unclear (1). Psychological assessment using the STAI reported significantly higher scores in the BMS patients compared with control subjects. However, no correlation between STAI score and pain ratings could be found. Previous studies, too, saw no association between pain ratings and psychological factors in BMS patients [27, 28]. Taken together, these results suggest that the contribution of anxiety to pain rating differences is limited.

### Limitations

We are aware of the limitations that apply to the present study. First, our sample was limited to females and our results thus cannot be generalized to males. Second, we studied a peri-menopausal population in which CPM effect is known to weaken with age [29]. Third, although the sample size was selected based on a previous study [12], the methodology in the present study did not exactly match that in the previous one, and consequently, the sample size might have been underestimated to detect between‐group differences. Finally, we did not look for possible differences between body regions [30].

## Conclusions

This study reveals that BMS patients’ sensory profile is characterized by enhanced extracephalic heat hyperalgesia, without alterations in CPM. Altogether, the results suggest that an increase in endogenous pain facilitation and no impairment in inhibition underlies BMS in women.

## Data Availability

The datasets used and/or analyzed during the current study are available from the corresponding author on reasonable request.

## Abbreviations

BDI: Beck depression inventory
BMS: Burning mouth syndrome
CPM: Conditioned pain modulation
HPT: Heat pain thresholds
MPT: Mechanical pain threshold
PCS: Pain catastrophizing scale
STAI: State-trait anxiety inventory
TSP: Temporal summation of pain
VAS: Visual analogue scale
VRS: Verbal rating scale
